# Inuloxin E, a New Seco-Eudesmanolide Isolated from *Dittrichia viscosa*, Stimulating *Orobanche cumana* Seed Germination

**DOI:** 10.3390/molecules24193479

**Published:** 2019-09-25

**Authors:** Marco Masi, Mónica Fernández-Aparicio, Roukia Zatout, Angela Boari, Alessio Cimmino, Antonio Evidente

**Affiliations:** 1 Dipartimento di Scienze Chimiche, Università di Napoli Federico II, Complesso Universitario Monte S. Angelo, Via Cintia 4, 80126 Napoli, Italy; marco.masi@unina.it (M.M.); roukia.zatout@umc.edu.dz (R.Z.); evidente@unina.it (A.E.); 2 Institute for Sustainable Agriculture, CSIC, Apdo. 4084, 14080 Córdoba, Spain; 3 Department of Applied Biology, University of Fréres Mentouri Constantine 1, Route Ain El Bey 25000, Constantine, Algeria; 4 Institute of Food Production Sciences, National Research Council, Via Amendola 122/O, 70125 Bari, Italy; angela.boari@ispa.cnr.it

**Keywords:** *Dittrichia viscosa*, *Orobanche cumana*, sesquiterpenoids, inuloxin E, inuloxin D derivatives, broomrapes seed germination stimulants

## Abstract

A new sesquiterpenoid belonging to the subgroup seco-eudesmanolides and named inuloxin E was isolated from *Dittrichia viscosa*, together with the already known sesquiterpenoids inuloxins A–D and α-costic acid. Inuloxin E was characterized by spectroscopic data (essentially NMR and ESI MS) as 3-methylene-6-(1-methyl-4-oxo-pentyl)-3a,4,7,7a-tetrahydro-3*H*-benzofuran-2-one. Its relative configuration was determined by comparison with the closely related inuloxin D and chemical conversion of inuloxin E into inuloxin D and by the observed significant correlation in the NOESY spectrum. Both inuloxins D and E induced germination of the parasitic weed *Orobanche cumana*, but were inactive on the seeds of *Orobanche minor* and *Phelipanche ramosa*. The germination activity of some hemisynthetic esters of inuloxin D was also investigated.

## 1. Introduction

The broomrapes (*Orobanche* and *Phelipanche* spp.) are obligate, chlorophyll-lacking, root parasitic weeds that obtain all their nutrient resources by attacking crops, inflicting high yield losses in many parts of Europe, Asia, and Africa. *Orobanche cumana* is one of the most virulent broomrape weeds that is highly specialized at attacking sunflowers. *Phelipanche ramosa* and *Orobanche minor* have broader host ranges, infecting not only many crops, but also their associated non-parasitic weeds and other wild plants. The underlying cause in such host range variation is often due to adaptation to the specific germination stimulants that are exuded by the roots of their hosts [1,2]. There are few effective control measures against these broomrape species. Current management of parasitic plants is primarily based on the use of preventive measures such as the use of resistant cultivars or the elimination of susceptible crops from the rotation. However, when the crop plant is already infected by these weeds, the use of herbicides is the only option. Resistant cultivars to broomrape infection are not commercially available for some crop species nor effective against some broomrape strains. Furthermore, crop rotation with non-host crops may not efficiently reduce the seed bank if the broomrape species can thrive on a broad range of plant species including other crops or non-parasitic weeds. Amino acid inhibiting herbicides are applied on the foliar system of infected crops and are translocated downwards through the haustorial connection, successfully targeting the underground parasitic weed. However, the repetitive use of traditional chemical herbicides can reduce the health value of crops and increase the risk of the emergence of herbicide resistances in the weed. Therefore, there is need for a diversification of efficient alternatives to conventional broomrape control in order to achieve sustainable plant protection programs [3]. An alternative to the use of traditional chemicals is the use of natural products-based herbicides. Fungal and plant metabolites have showed their ability to stimulate or inhibit broomrape seed germination and the activity that resulted was compound and broomrapes species dependent [4]. Inuloxins A–D and α-costic acid, isolated from *Dittrichia viscosa* (syn of *Inula viscosa*), a native and spontaneous Mediterranean plant, have demonstrated the ability to inhibit *Orobanche crenata* and *Cuscuta campestris* seed germination [5]. Successively, inuloxin A and α-costic acid induced significant germination of *O. cumana* seeds while inuloxin C induced a phytotoxic effect on four broomrapes as the radicles exposed to this compound were shorter and had light brown patches [4]. Considering that the absolute configuration (AC) of naturally occurring compounds is closely related to their biological activity [6,7], the AC of inuloxin A, the main germacrane sesquiterpene isolated from *D. viscosa*, was determined by chiroptical and computational methods [8]. The same methods were successively used to assign the AC to the eudesmanolide inuloxin C [9], while studies are in progress to assign that of seco-eudesmanolide inuloxin D. These results prompt a re-investigation of *D. viscosa* organic extracts to find new inuloxins with potential herbicidal activity and to carry out studies on the structure activity relationships of this class of natural compounds. This manuscript reports the isolation and the chemical and biological characterization of a new inuloxin, named inuloxin E, the activity of both inuloxins D and E in *O. cumana*, *O. minor*, and *P. ramosa* seed germination, and radicle development. Furthermore, the specific activity in stimulating *O. cumana* seed germination of some hemisynthesized derivatives of inuloxin D was also described.

## 2. Results and Discussion

**Figure 1:**
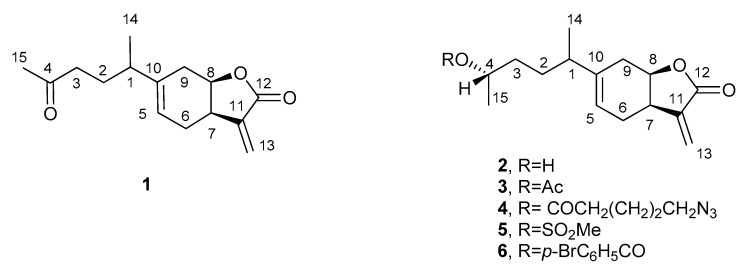
The structures of inuloxin E (**1**), inuloxin D (**2**), and inuloxin D derivatives (**3**–**6**).

The extract obtained from the aerial part of *D. viscosa* was purified, as reported in detail in the material and methods section, to afford α-costic acid, inuloxins A, C, and D (**2**, Figure 1) and the new sesquitepenoid, named inuloxin E (**1**, Figure 1).

Inuloxin E had a molecular formula of C_15_H_20_O_3_ as deduced from its HR ESIMS spectrum, which is consistent with six hydrogen deficiencies. The preliminary ^1^H and ^13^C-NMR investigation showed that it was very close to inuloxin D, however it differed as it lacked two hydrogens. Its ^1^H-NMR spectrum (Table 1) showed the signal pattern of pentahydrobenzofuranone moiety as in **2**. In fact, the protons of an exocyclic methylene and that of a trisubstituted olefinic group resonating as two doublets (*J* = 3.2 Hz) and a broad double doublet (*J* = 8.8 and 5.3 Hz) at δ 6.28 and 5.54 and 5.46, respectively [10].

The latter is coupled in the COSY spectrum [11] with the two protons of the adjacent methylene group (H_2_C-6) which appeared as two multiplets at δ 2.43 and 2.18 also being coupled with the proton of the adjacent methine carbon (HC-7) appearing as a multiplet at δ 3.34. Being the proton of one of the head-bridge carbons of the junction between the cyclohexene and the furanone ring, H-7 was coupled with the proton (H-8) of the other head-bridge carbon (C-8), which resonated as a doublet of double doublets (*J* = 11.8, 8.4 and 2.7 Hz) at δ 4.66. H-8 also coupled with the protons of the adjacent methylene group (H_2_C-9), appearing both as a doublet of double doublets (*J* = 13.0, 6.0 and 2.7 and 13.0, 11.8 and 8.4 Hz) at δ 2.02 and 1.89. The signals of the side chain significantly differed from those of inuloxin D. The signals of H-1 and H_2_-2 appeared as multiplets at δ 2.37 and 2.26, which is very similar to the value observed in **2** as well as the doublet (*J* = 6.9 Hz) of the secondary methyl group (Me-14), which resonated at δ 1.16. Instead, a significant downfield shift (Δδ 1.04 and 0.78) was observed for the protons of C-3 resonating as two multiplets at δ 2.56 and 2.26, and the signal of H-4 is missing. Finally, the terminal methyl group (Me-15) appeared as a singlet downfield shifted (Δδ 0.93) at δ 2.17 [10]. These finding are consistent with a 1-methyl-4-oxopentyl side chain attached at C-10 of the pentahydrobenzobenzofuranone of **1** as also supported from the HMBC couplings observed in the HMBC spectrum [11] (Table 1) between C-1 with H-5, H_2_-6 and H_2_-9, and between C-10 with H-1 and Me-14. The couplings observed in the HSQC spectrum [11] (Table 1) allowed us to assign the chemical shifts to all the protonated carbons and, in particular, the signals at δ 122.1, 120.1, 79.3, 42.6, 42.1, 36.7, 35.4, 30.4, 29.9, 26.6, and 20.9 were assigned to C-13, C-5, C-8, C-3, C-7, C-9, C-1, C-2, C-15, C-6, and C-14. [12] The ^13^C-NMR spectrum (Table 1) also showed the signals of one ketone and one lactone carbonyls at δ 208.0 (C-4) and 170.2 (C-12) and those of two quaternary olefinic carbons at δ 144.5 (C-10) and 139.0 (C-11), which were assigned by the correlations, observed in the HMBC spectrum between C-4 with H_2_-3 and Me-15, C-12 and H-7, H-8 and H_2_-13, C-10 and H-1, H_2_-6, H_2_-9, and Me-14, and C-11 with H_2_-6, H-7, H-8, and H_2_-13.

**Figure 2:**
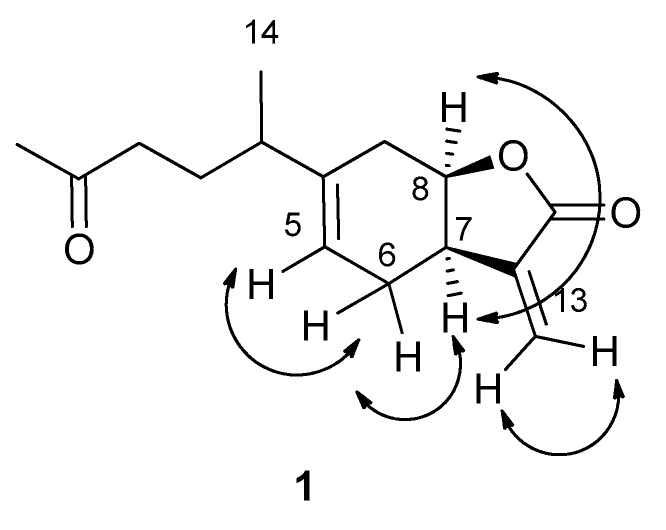
Correlations observed in the NOESY spectrum of inuloxin E (**1**).

Thus, the chemical shifts to all the protons and corresponding carbons of **1** were assigned, as reported in Table 1, and inuloxin E was formulated as 3-methylene-6-(1-methyl-4-oxo-pentyl)-3a,4,7,7a-tetrahydro-3*H*-benzofuran-2-one. The structure that was assigned to **1** was further supported by the absence of any signal due to the hydroxy group in the IR spectrum and by the other couplings observed in the HMBC spectrum and reported in Table 1. The HR ESIMS spectrum showed that it sodiated [M + Na]^+^ at *m*/*z* 271.1321, while its ESIMS showed the sodiated dimeric cluster and protonated [M + H]^+^ forms at *m*/*z* 519 and 249, respectively. Considering the value (*J* = 8.4 Hz) for the coupling constant measured between H-7 and H-8 in comparison with the same value recorded in **2**, a *cis*-junction could be attributed between the two rings of the pentahydrobenzofurane moiety. This was strongly confirmed by the data obtained from the NOESY experiment [11] (Figure 2).

In fact, in the NOESY spectrum, the significant correlation between H-7 and H-8 was observed together with the expected coupling between the two protons of the exocyclic olefinic methylene group H_2_C-13 and the protons of H_2_C-6a with both H-5 and H-7. A further support to the structure assignment of inuloxin E was obtained by the reduction of **1** with sodium borohydride. The main product obtained was identified as inuloxin D by comparison of its spectroscopic and optical rotation data with those previously reported for the natural **2** [5], suggesting that there was stereoselectivity in the reduction reaction. These results were not a surprise as stereoselective reduction of the ketone group of some naturally occurring compounds with sodium borohydride was already observed [13,14,15,16,17].

Inuloxins E and D were assayed in comparison with four different ester derivatives prepared from inuloxin D (**3**–**6**). In particular, the acetyl derivative of **2** (**3**) was prepared by usual acetylation and its ^1^H-NMR spectrum (Table 2) differed from that of the parent compound. In particular, for the downfield shift (Δδ 1.04) of H-4, resonating as a multiplet at δ 4.84, and the presence of the singlet of the acetyl group at δ 2.06. Its ESI MS showed the sodiated [M + Na]^+^ and protonated [M + H]^+^ forms at *m/z* 315 and 293. The 4-*O*-azidopentanoyl ester of **2** (**4**), prepared by dehydratation between **2** and the 5-azidopentanoyl acid, showed a ^1^H-NMR spectrum (Table 2), which differed from that of **2** for the downfield shift (Δδ 1.10) of H-4, resonating as a multiplet at δ 4.90, and for the signal system of the azidopentanoyl residue observed at δ 2.36 (t, *J* = 6.9 Hz, CH_2_-2′), 1.79–1.62 (CH_2_-3′ and CH_2_-4′), and 3.33 (t, *J* = 6.9 Hz, CH_2_-5′). The ESI-MS spectrum showed the sodiated [M + Na]^+^ and protonated [M + H]^+^ forms at *m/z* 398 and 376. The 4-*O*-mesyl ester of **2** (**5**) was prepared by the reaction of inuloxin D with mesyl chloride. Its ^1^H-NMR spectrum (Table 2) differed from that of **2** for the downfield shift (Δδ 1.02) of H-4, resonating as a multiplet at δ 4.82, and the presence of the singlet of the mesyl group at δ 3.04. Its ESI MS showed the sodiated [M + Na]^+^ and protonated [M + H]^+^ forms at *m/z* 351 and 329. Finally, the *p*-bromobenzoyl ester of **2** (**6**) was prepared by the reaction between inuloxin D and *p*-bromobenzoyl chloride. The ^1^H-NMR spectrum (Table 2) differed from that of **2** or the downfield shift (Δδ 1.30) of H-4, resonating as a multiplet at δ 5.10 and the presence of the two doublets (*J* = 8.7 Hz) of the *p*-Br-substituted benzoyl residue at δ 7.90 and 7.59. Its HR ESI MS showed the typical sodiated [M + Na]^+^ and protonated [M + H]^+^ forms at *m/z* 454 and 452 and 432 and 430, respectively.

**Figure 3:**
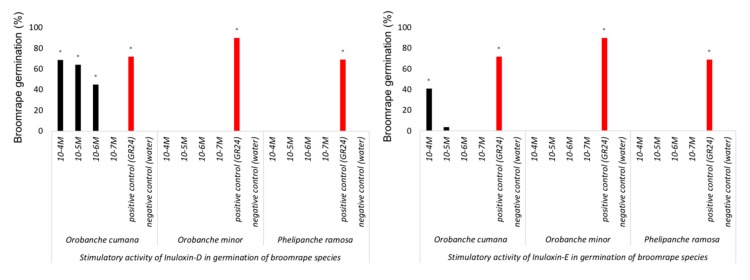
*Orobanche cumana*, *Orobanche minor*, and *Phelipanche ramosa* germination induced by inuloxin D (left panel) and inuloxin E (right panel). The asterisk (*) indicates differences at the 0.05 level compared with the negative control (seeds induced to germinate with water).

The stimulation activity of inuloxins D and E was studied in three species of broomrape weeds: *O. cumana*, *O. minor*, and *P. ramosa* (Figure 3).

None of those inuloxins were active inducing germination of *O. minor* and *P. ramosa*, two broomrape species known for their low level of specialization in root exudate recognition [1,2]. On the contrary, both inuloxins induced germination of the highly selective seeds of *O. cumana*. In *O. cumana*, the effect of inuloxin D was stronger than that induced by inuloxin E and, therefore, four ester derivatives, as reported above, were prepared from inuloxin D and tested in all broomrape species (Table 3).

The structural modifications made in inuloxin D did not carry induction activity on *O. minor* and *P. ramosa* seeds inducing their germination. The germination level of *O. cumana* was reduced in **4** and **6** at all concentrations tested in comparison with inuloxin D. At 10^−4^ M, the levels of *O. cumana* germination induced by **3** and **5** were comparable with that induced by **2**, however, at lower concentrations, the germination activity was reduced in these derivatives in comparison with inuloxin D.

**Figure 4:**
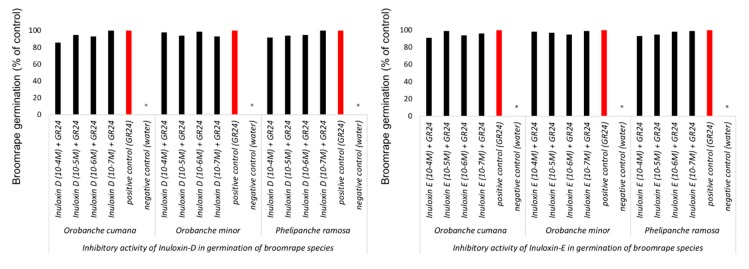
Inhibition of *Orobanche cumana*, *Orobanche minor*, and *Phelipanche ramosa* germination tested by the application of GR24 alone or GR24 mixed with inuloxin D (left panel) and inuloxin E (right panel). Data is expressed as the percentage referred to as the control GR24. The asterisk (*) indicates the differences at the 0.05 level compared with the positive control (seeds induced to germinate with only GR24).

**Figure 5:**
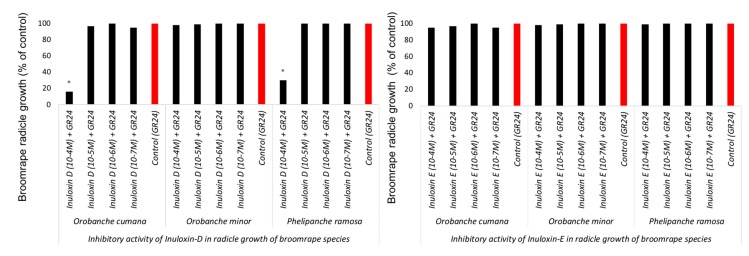
*Orobanche cumana*, *Orobanche minor*, and *Phelipanche ramosa* radicle growth in the presence of GR24 alone or GR24 mixed with inuloxin D (left panel) and inuloxin E (right panel). Data is expressed as the percentage referred to as the control GR24. The asterisk (*) indicates the differences at the 0.05 level compared with the positive control (GR24).

The inhibition activity of inuloxin E and inuloxin D in the seeds of three species of broomrape weeds: *O. cumana*, *O. minor*, and *P. ramosa* was studied by mixing these metabolites with GR24, a synthetic germination stimulant that is active in the three broomrape weeds that were studied in this work. No inhibitory action was observed in inuloxin D and inuloxin E as the germination activity of GR24 that was mixed with each inuloxin was not different from that induced by the GR24 control. This effect was observed for *O. cumana* and also for the species *O. minor* and *P. ramosa* and, therefore, confirmed that the effect reported in Figure 4 for *O. minor* and *P. ramosa* is a lack of stimulatory activity and not inhibitory action. Interestingly, *O. cumana* and *P. ramosa* seeds that germinated with GR24 mixed with inuloxin D developed shorter radicles (Figure 5). This inhibition of radicle growth was not observed in seeds of *O. minor*. Inuloxin E did not inhibit the radicle growth in any of the broomrape species tested.

## 3. Materials and Methods

### 3.1. General Experimental Procedures

Optical rotation was measured in CHCl_3_ solution by a P-1010 digital polarimeter (Jasco, Tokyo, Japan). IR spectra were recorded as deposit glass film on a Thermo Nicolet 5700 FT-IR spectrometer (Madison, WI, USA). UV spectra were measured in MeOH on a V-530 spectrophotometer (Jasco, Tokyo, Japan). ^1^H and ^13^C-NMR spectra were recorded at 400 and 100 MHz, respectively, in CDCl_3_ by Bruker spectrometers (Karlsruhe, Germany). The same solvent was used as the internal standard. Carbon multiplicities were determined by DEPT spectra [11]. DEPT, COSY-45, HSQC, HMBC, and NOESY experiments [10] were performed using Bruker microprograms. HR ESIMS and ESIMS were recorded using the LC/MS ESIMS-TOF system (Agilent 6230B, HPLC 1260 Infinity) (Milan, Italy). The HPLC separations were performed using a Phenomenex LUNA (C18 (2) 5u 150 × 4.6 mm) (Torrance, CA, USA). Analytical, preparative, and reverse phase TLCs were carried out on silica gel (Merck, Kieselgel 60, F254, 0.25, 0.5 mm, and RP-18 F254s, respectively) plates (Merck, Darmstadt, Germany). The spots were visualized by exposure to UV radiation or by spraying them first with 10% H_2_SO_4_ in MeOH and then with 5% phosphomolybdic acid in EtOH, followed by heating at 110 °C for 10 min. Column chromatography was performed using silica gel (Kieselgel 60, 0.063–0.200 mm) (Merck, Darmstadt, Germany).

### 3.2. Plant Material

Whole aerial parts of *Dittrichia viscosa* plants were collected fresh in Southern Italy. A voucher specimen was deposited at the Collection of Istituto di Scienze delle Produzioni Alimentari, CNR, Bari, Italy. After harvesting, the leaves were detached from the stems and were dried in a ventilated oven at 50 °C for two days. The plant material was then grinded to obtain a tiny powder by using a laboratory mill and was packaged in plastic bags under vacuum until its use. The broomrape weeds assayed were *Orobanche cumana* collected from sunflowers in Spain, *Orobanche minor* collected from red clover in France, and *Phelipanche ramosa* collected from tobacco in France.

### 3.3. Isolation of Plant Metabolites

Plant material (450 g) was extracted (1 × 1 L) by H_2_O/MeOH (1/1, *v*/*v*) under stirred conditions at room temperature for 24 h, and the suspension was centrifuged and the supernatant was extracted by CH_2_Cl_2_ (3 × 400 mL), as previously reported [5]. The organic extract (17 g) was purified by column chromatography on silica gel and was eluted with CHCl_3_/*i*-PrOH (95/5, *v*/*v*), yielding nine groups of homogeneous fractions. The residue of the second and third fractions were combined and further purified, as previously reported [5], to get the main metabolites α-costic acid and inuloxin A. The residue (1.1 g) of the fifth fraction was further purified under the same conditions reported above, giving seven homogeneous fraction groups. The residue (36.8 mg) of the first fraction of the latter column was purified by preparative TLC using EtOAc/*n*-hexane (6/4, *v*/*v*), giving both inuloxins E and D as pure oils (**1**, Rf 0.65, 11.6 mg, and **2**, Rf 0.48, 5.8 mg) and inuloxin C (Rf 0.40, 11.0 mg).

#### 3.3.1. Inuloxin E (**1**)

[α]_D_^25^ + 21.4 (c 0.4); IR ν_max_ 1762, 1714, 1660 cm^−1^; UV λ_max_ nm (log ε) end absorbtion. ^1^H and ^13^C-NMR data: see Table 1; HR ESIMS (+), *m/z* 271.1321 (C_15_H_20_NaO_3_, calcd 271.1310 [M + Na]^+^); ESIMS (+), *m*/*z* 519 [2M + Na]^+^, 249 [M + H]^+^. ^1^H and ^13^C-NMR, HSQC, HMBC, COSY, NOESY, and IR spectra are available as Supplementary Material.

#### 3.3.2. NaBH_4_ Reduction of Inuloxin E (**1**)

Inuloxin E (**1**, 2.0 mg), dissolved in MeOH (1.0 mL), was added to NaBH_4_ (2 mg) and the reaction was performed under stirring at room temperature for 30 min. The mixture was neutralized with 0.1 M HCl, extracted with CH_2_Cl_2_ (3 × 30 mL), and dried (Na_2_SO_4_). The oily residue was purified by TLC, using petroleum ether/acetone (8/2, *v*/*v*) for elution to give the main metabolite inuloxin D (**2**) as an oil (0.8 mg, Rf 0.37). Inuloxin D had spectroscopic and optical data similar to those previously reported in literature [5].

### 3.4. Preparation of Hemisynthetic Derivatives of Inuloxin D

#### 3.4.1. 4-*O*-Acetyl Derivative of Inuloxin D (**3**)

Inuloxin D (**2**, 2 mg), dissolved in pyridine (40 µL), was converted in its corresponding acetyl ester (**3**) by acetylation with Ac_2_O (40 µL). The reaction was carried out under stirring for 1 h at room temperature. It was stopped with MeOH and the azeotrope that was formed by the addition of C_6_H_6_ was evaporated under nitrogen stream. The residue was then purified by TLC eluted with CHCl_3_/*i*-PrOH (98/2, *v*/*v*) yielding **3** (1.2 mg) as an amorphous oil. Derivative **3** had: Rf 0.75; ^1^H-NMR data: see Table 2; ESIMS (+), *m/z* 315 [M + Na]^+^, 293 [M + H]^+^.

#### 3.4.2. 4-*O*-Azidopentanoyl Ester of Inuloxin D (**4**)

Then, 5.0 mg of DCC and 15 µL of 5-azidopentanoic acid were added to a solution of inuloxin D (**2**, 2.0 mg) in pyridine (100 µL). The mixture was kept under stirring at room temperature for 24 h and the reaction was stopped by the addition of C_6_H_6_ and MeOH, and was dried under nitrogen stream. The residual oil was then purified by TLC eluted with CHCl_3_, affording 5-azidopentanoyl ester of inuloxin D (**4**, 1.5 mg). Derivative **4** had: Rf 0.70; ^1^H-NMR data: see Table 2; ESIMS (+) *m/z* 398 [M + Na]^+^, 376 [M + H]^+^.

#### 3.4.3. 4-*O*-Mesyl Ester of Inuloxin D (**5**)

Mesyl chloride (25 µL) was added to inuloxin D (**2**, 2.0 mg), which was dissolved in CH_2_Cl_2_ (300 mL) and pyridine (40 µL). The reaction was carried out for 2 h at room temperature. It was stopped with MeOH, and the azeotrope that was formed by the addition of C_6_H_6_ was evaporated under nitrogen stream. The residue was then purified by TLC eluted with CHCl_3_ yielding **5** (1.2 mg) as an amorphous oil. Derivative **5** had: Rf 0.72; ^1^H-NMR data: see Table 2; ESIMS (+) *m/z* 351 [M + Na]^+^, 329 [M + H]^+^.

#### 3.4.4. 4-*O*-*p*-Bromobenzoylester of Inuloxin D (**6**)

DMAP (5.0 mg) and *p*-bromobenzoyl chloride (5.0 mg) were added to inuloxin D (**2**, 2.0 mg), which was dissolved in anhydrous MeCN (100 µL). The reaction mixture was left under stirring for 4 h. It was then quenched with a 1N NaHCO_3_ and extracted with CH_2_Cl_2_. The residue obtained after solvent evaporation was purified by preparative TLC and was eluted with CHCl_3_ to afford the *p*-bromobenzoyl ester of inuloxin D (**6**, 0.9 mg). Derivative **6** had: Rf 0.76; ^1^H-NMR data: see Table 2; ESIMS (+) *m/z* 454 and 452 [M + Na]^+^ 432 and 430 [M + H]^+^.

### 3.5. Germination Induction Bioassays

The germination activity of inuloxin D, inuloxin E, and inuloxin D derivatives was assayed on *O. cumana*, *O. minor*, and *P. ramosa* seeds. The seeds were surface sterilized by immersion in 0.5% (*w*/*v*) NaOCl and 0.02% (*v*/*v*) Tween 20 and sonication for 2 min, and were then rinsed thoroughly with sterile distilled water and dried in a laminar air flow cabinet. Approximately 100 seeds of each broomrape species were placed separately on 9 mm diameter glass fiber filter paper discs (GFFP) moistened with 50 μL of sterile distilled water. Subsequently, GFFP discs were placed inside sterile 10 cm Petri dishes, sealed with parafilm, and conditioned for 7 days at 22 °C in the dark. Tested samples, dissolved in acetone, were diluted with sterilized distilled water to a final concentration of 10^−4^ M. The final concentration of acetone was adjusted to 0.70% (*v*/*v*). GFFP discs containing conditioned seeds were transferred inside laminar flow cabinet to sterile filter paper to remove excess water, and were then transferred to a new 10 cm sterile Petri dish. For each broomrape species, 50 μL-aliquots of each test solution was applied to three discs containing conditioned seeds. Seeds treated with distilled water (containing 0.70% acetone) or the synthetic strigolactone GR24 were included as controls for comparison. The seeds were incubated in the dark at 22 °C for 7 days prior to examination for germination. Seeds with an emerged radicle were scored as germinated using a stereoscopic microscope at 30× magnification, and the percentage of germination was established for each dish.

### 3.6. Germination and Growth Inhibition Bioassays

The inhibitory activity of inuloxins D and E on seed germination and radicle growth of *O. cumana*, *O. minor*, and *P. ramosa* was assayed as reported previously [18]. A solution of GR24 (10^−6^ M) was prepared in sterile distilled water. Immediately before use, the stock solutions of each inuloxin prepared in acetone were diluted in the GR24 solution to 10^−4^ M of each toxin while keeping the GR24 concentrations and acetone constant in order to allow for comparisons. The broomrape seeds were surface sterilized and conditioned, as described above. Fifty microliter aliquots of each inuloxin-GR24 were applied to each GFFP disc containing the conditioned seeds. Petri dishes were sealed with Parafilm and stored in the dark at 22 °C for 7 days to promote germination and radicle growth. The percentage of seed germination and radicle length were established for each GFFP disc in order to score levels of inuloxin-mediated inhibition. The germination percentage was determined by scoring the number of seeds with an emerged radicle through the seed coat in a total of 100 seeds per disc. Radicle length was measured in 15 randomly selected germinated seeds from each disc.

### 3.7. Statistical Analysis

The bioassays were performed twice with three replicates. Percentage data were approximated to normal frequency distribution by means of angular transformation (180/π × arcsine (sqrt [%/100])) and were subjected to analysis of variance (ANOVA) using SPSS software for Windows, version 21.0 (SPSS Inc., Chicago, IL, USA). The significance of the mean differences between each treatment against its respective control was evaluated by the two-sided Dunnett test. The null hypothesis was rejected at the level of 0.05.

## 4. Conclusions

A new seco-eudesmanolide named inuloxin E was isolated from *D. viscosa* and was chemically and biologically characterized. Although *D. viscosa* plant extract showed the presence of several classes of natural metabolites and a wide range of bioactivities, which were also recently reviewed, [19] the isolation of the new inuloxin E suggests that further bioactive metabolites could be discovered from this plant. Both inuloxins D and E induced germination of the parasitic weed *O. cumana*, but they were inactive on seeds of *O. minor* and *P. ramosa*. This selective activity is important from a practical point of view as both inuloxins D and E could be used to develop a so called “suicidal germination” method [20,21,22] for the specific control of the parasitic weed *O. cumana*. This is a valuable alternative for the control of a noxious parasitic weed based on a suitable formulation of plant metabolites that could be obtained in large amount. In fact, *D. viscosa* could be cultivated for the development of a convenient and friendly environmental synthesis of both inuloxins.

## Figures and Tables

**Table 1:** ^1^H and ^13^C-NMR data of inuloxin E (**1**) ^*a*,*b*^.

Position	δC *^c^*	δH (*J* in Hz)	HMBC
1	35.4 d	2.37 m	H_2_-6, H-5, H_2_-9, H-8, Me-14
2	30.4 t	2.26 m (2H)	H-3A, H-5
3	42.6 t	2.56 m 2.26 m	H_2_-2
4	208.0 s		H_2_-3, Me-15
5	120.1 d	5.46 br dd (8.8, 5.3)	H_2_-6
6	26.6 t	2.43 m 2.18 m	H-5, H-7, H-8
7	42.1 d	3.34 m	H-5, H_2_-6, H-8, H_2_-9, H_2_-13
8	79.3 d	4.66 ddd (11.8, 8.4, 2.7)	H_2_-6, H-7, H_2_-9
9	36.7 t	2.02 ddd (13.0, 6.0, 2.7) 1.89 ddd (13.0, 11.8, 8.4)	H-1, H-7
10	144.5 s		H-1, H_2_-6, H_2_-9, Me-14
11	139.0 s		H_2_-6, H-7, H-8, H_2_-13
12	170.2 s		H-7, H_2_-13, H-8
13	122.1 t	6.28 d (3.2) 5.54 d (3.2)	H-7
14	20.9 q	1.16 d (6.9)	H_2_-9, H-1, H-5
15	29.9 q	2.17 s	

*^a^* The chemical shifts are in δ values (ppm) from TMS. *^b^* 2D ^1^H, ^1^H (COSY) ^13^C, ^1^H (HSQC) NMR experiments delineated the correlations of all the protons and the corresponding carbons. *^c^* Multiplicities were assigned by the DEPT spectrum.

**Table 2:** ^1^H-NMR data of inuloxin D (**2**) and its derivatives (**3**–**6**).

Position	2 *^a^*	3 *^b^*	4 *^c^*	5 *^d^*	6 *^e^*
	δH, (*J* in Hz)	δH, (*J* in Hz)	δH, (*J* in Hz)	δH, (*J* in Hz)	δH, (*J* in Hz)
1	2.39 m	2.36 sextet (6.6)	2.40 m	2.36 sextet (6.6)	2.36 sextet (6.9)
2	2.06 m 2.04 m	2.06 (2H) m	2.06 (2H) m	2.05 (2H) m	2.06 (2H) m
3	1.52 m 1.48 m	1.74 m 1.53 m	1.79 m 1.62 m	1.86 m 1.66 m	1.76 m 1.65 m
4	3.80 m	4.84 m	4.90 m	4.82 m	5.10 m
5	5.52 dd (8.7, 4.9)	5.47 dd (9.2, 5.2)	5.46 dd (9.2, 5.4)	5.54 dd (9.2, 5.2)	5.42 dd (8.9, 5.4)
6	2.48 m 2.21 m	2.45 br dd (14.2, 5.2) 2.19 ddd (14.2, 9.2, 4.5)	2.45 br dd (13.8, 5.4) 2.19 ddd (13.8, 9.2, 4.5	2.47 br dd (13.8, 5.2) 2.22 ddd (13.8, 9.2, 5.4)	2.42 br dd (14.2, 5.4) 2.13 ddd (14.2, 8.9, 4.6)
7	3.36 m	3.38 m	3.38 m	3.38 m	3.35 m
8	4.66 ddd (11.8, 8.6, 2.9)	4.67 ddd (11.8, 8.3, 2.7)	4.67 ddd (11.5, 7.7, 2.4)	4.46 ddd (11.8, 8.6, 2.5)	4.66 ddd (11.6, 8.8, 2.5)
9	2.01 m 1.98 m	1.96 (2H) m	1.96 (2H) m	1.96 (2H) m	2.01 m 1.93 m
13	6.27 d (3.2) 5.55 d (3.2)	6.28 d (2.9) 5.55 d (2.9)	6.28 d (2.9) 5.55 d (2.9)	6.29 d (2.9) 5.56 d (2.9)	6.26 d (3.0) 5.51 d (3.0)
14	1.16 d (6.9)	1.15 d (6.6)	1.15 d (6.7)	1.16 d (6.9)	1.12 d (6.9)
15	1.24 d (6.2)	1.25 d (6.2)	1.25 d (6.4)	1.46 d (6.1)	1.35 d (6.6)

*^a^* These data are the same as already reported in Andolfi et al. 2013; *^b^* the singlet of the acetyl group was present at δ 2.06; *^c^* the signal system of the azidopentanoyl residue were observed at δ 2.36 (t, *J* = 6.9 Hz, CH_2_-2′), 1.79-1.62 (CH_2_-3′ and CH_2_-4′), 3.33 (t, *J* = 6.9 Hz, CH_2_-5′); *^d^* the singlet of the mesyl group resonated at δ 3.04; *^e^* the two doublets (*J* = 8.7) of the *p*-Br-substituted benzoyl residue were observed at δ 7.90 and 7.59.

**Table 3:** The induction of seed germination of three broomrape species *O. cumana*, *O. minor*, and *P. ramosa* by inuloxin D derivatives (**3**–**6**).

Broomrape Seed Germination (%)				
Inuloxin D derivative	Concentracion	*O. cumana*	*O. minor*	*P. ramosa*
**3**	10^−4^ M	57.8	0.0	0.0
	10^−5^ M	14.9	0.0	0.0
	10^−6^ M	2.3	0.0	0.0
	10^−7^ M	0.0	0.0	0.0
**4**	10^−4^ M	64.7	0.0	0.0
	10^−5^ M	29.4	0.0	0.0
	10^−6^ M	4.2	0.0	0.0
	10^−7^ M	0.0	0.0	0.0
**5**	10^−4^ M	26.4	0.0	0.0
	10^−5^ M	0.0	0.0	0.0
	10^−6^ M	0.0	0.0	0.0
	10^−7^ M	0.0	0.0	0.0
6	10^−4^ M	18.6	0.0	0.0
	10^−5^ M	3.5	0.0	0.0
	10^−6^ M	0.0	0.0	0.0
	10^−7^ M	0.0	0.0	0.0
**GR24**	1	64.0	80.4	68.1
**Control**	0	0.0	0.0	0.0

## Supplementary Materials

The following are available online at https://www.mdpi.com/1420-3049/24/19/3479/s1, ^1^H and ^13^C-NMR, HSQC, HMBC, COSY, NOESY, and IR spectra.
