# Supplementary material for: Inuloxin E, a New Seco-Eudesmanolide Isolated from Dittrichia viscosa, Stimulating Orobanche cumana Seed Germination
Source: Molecules. 2019 Sep 25;24(19):3479. doi: 10.3390/molecules24193479 (PMC6803869; doi:10.3390/molecules24193479)
Supplement: Supplementary file 1 [file molecules-24-03479-s001.pdf]

Supporting Information

Inuloxin E, a New Seco-Eudesmanolide Isolated from *Dittrichia viscosa*, Stimulating *Orobanche cumana* Seed Germination

Marco Masi [1], Mónica Fernández-Aparicio [2,*], Roukia Zatout [1,3], Angela Boari [4], Alessio Cimmino [1,*] and Antonio Evidente [1]

1 Dipartimento di Scienze Chimiche, Università di Napoli Federico II, Complesso Universitario Monte S. Angelo, Via Cintia 4, 80126 Napoli, Italy; marco.masi@unina.it (M.M.); roukia.zatout@umc.edu.dz (R.Z.); evidente@unina.it (A.E.)
2 Institute for Sustainable Agriculture, CSIC, Apdo. 4084, 14080 Córdoba, Spain
3 Department of Applied Biology, University of Fréres Mentouri Constantine 1, Route Ain El Bey 25000, Constantine, Algeria
4 Institute of Food Production Sciences, National Research Council, Via Amendola 122/O, 70125 Bari, Italy; angela.boari@ispa.cnr.it
* Correspondence: monica.fernandez@ias.csic.es (M.F.-A.); alessio.cimmino@unina.it (A.C.); Tel.: +34-957499170 (M.F.-A.); +39-0812532126 (A.C.)

Academic Editors: Stefano Dall'Acqua and Vincenzo De Feo
Received: 26 July 2019; Accepted: 24 September 2019; Published: 25 September 2019

Brief Statement in nonsentence format listing the content of the material supplied

The content of the material supplied as Supporting Information is reported in the following list:

Supporting Information List

Spectra 1. ^{1}H NMR spectrum of inuloxin E (**1**) ($CDCl_3$, 400 MHz)

Spectra 2. ^{13}C NMR spectrum of inuloxin E (**1**) ($CDCl_3$, 100 MHz)

Spectra 3. HSQC spectrum of inuloxin E (**1**) ($CDCl_3$, 400/100 MHz)

Spectra 4. HMBC spectrum of inuloxin E (**1**) ($CDCl_3$, 400/100 MHz)

Spectra 5. COSY spectrum of inuloxin E (**1**) ($CDCl_3$, 100 MHz)

Spectra 6. NOESY spectrum of inuloxin E (**1**) ($CDCl_3$, 100 MHz)

Spectra 7. IR spectrum of inuloxin E (**1**)

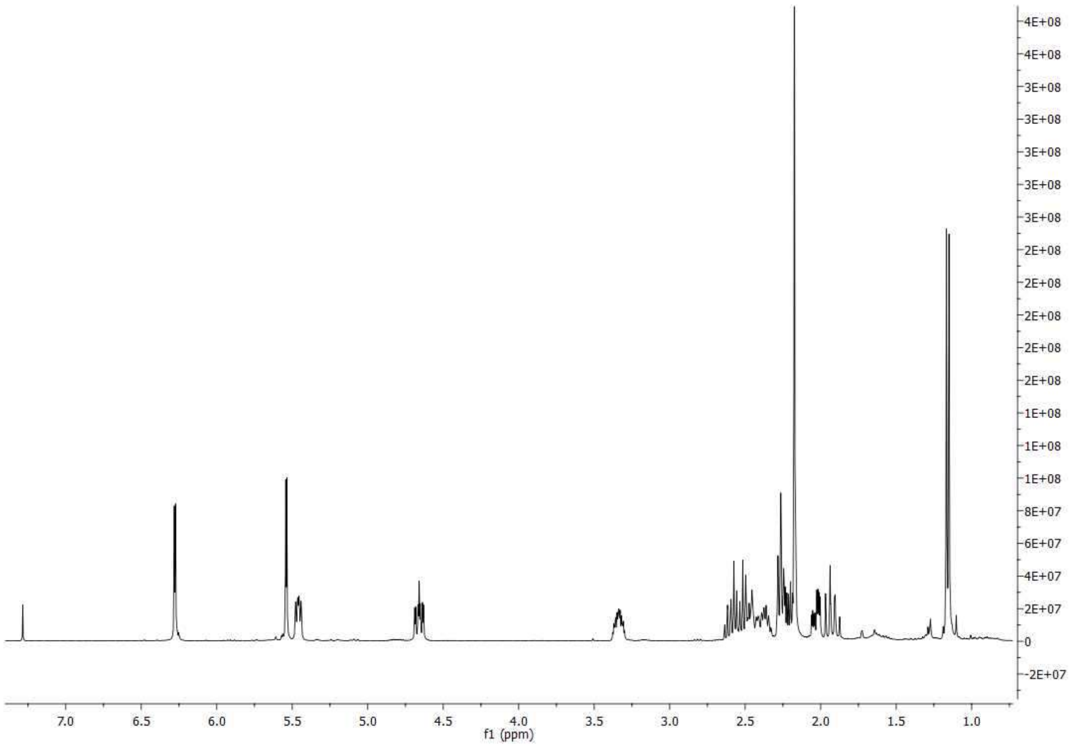

Spectra 1. ^{1}H NMR spectrum of inuloxin E (**1**) ($CDCl_3$, 400 MHz).

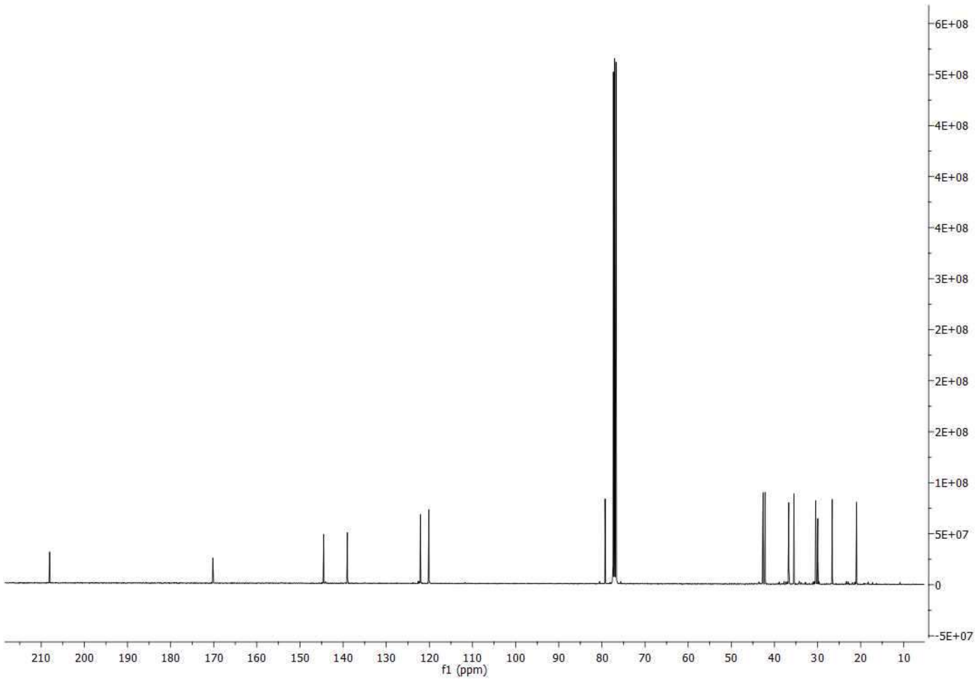

Spectra 2. ^{13}C NMR spectrum of inuloxin E (**1**) ($CDCl_3$, 100 MHz).

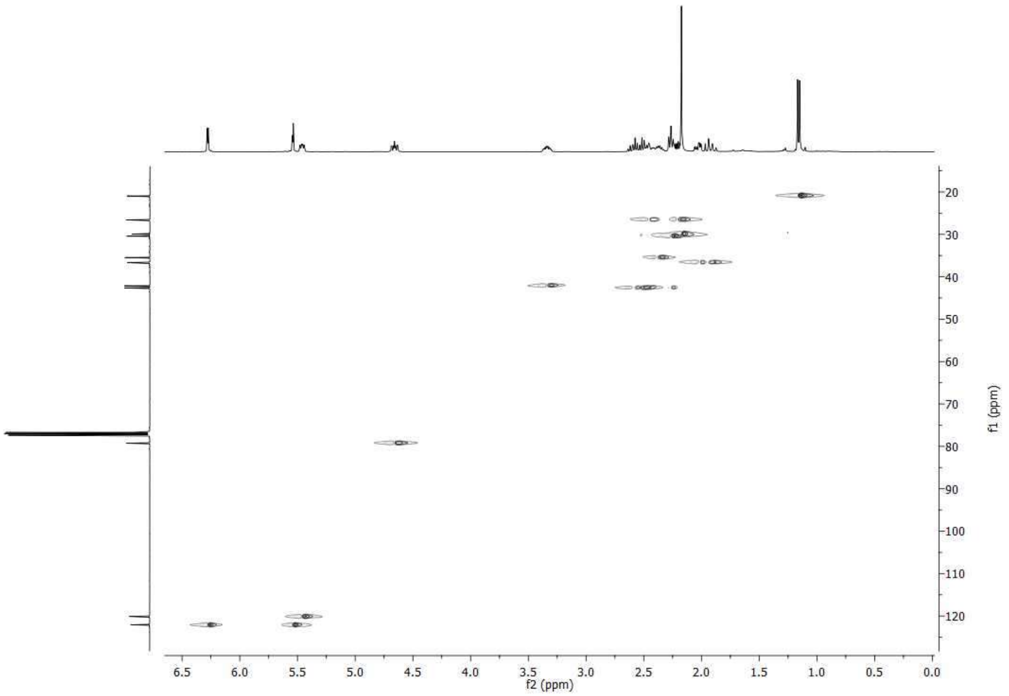

Spectra 3. HSQC spectrum of inuloxin E (**1**) ($CDCl_3$, 400/100 MHz).

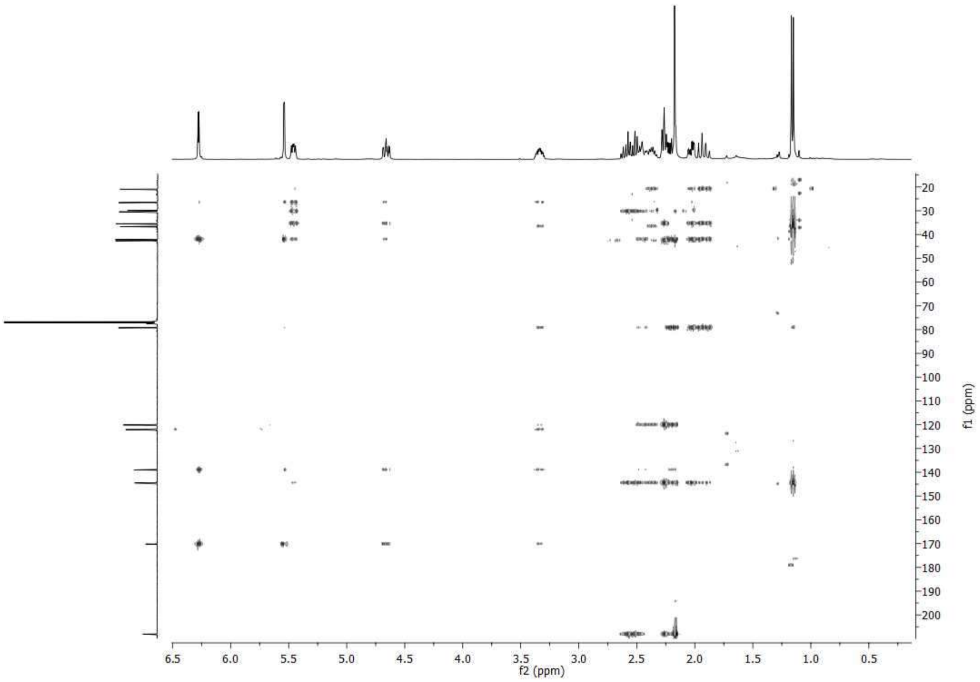

Spectra 4. HMBC spectrum of inuloxin E (**1**) ($CDCl_3$, 400/100 MHz).

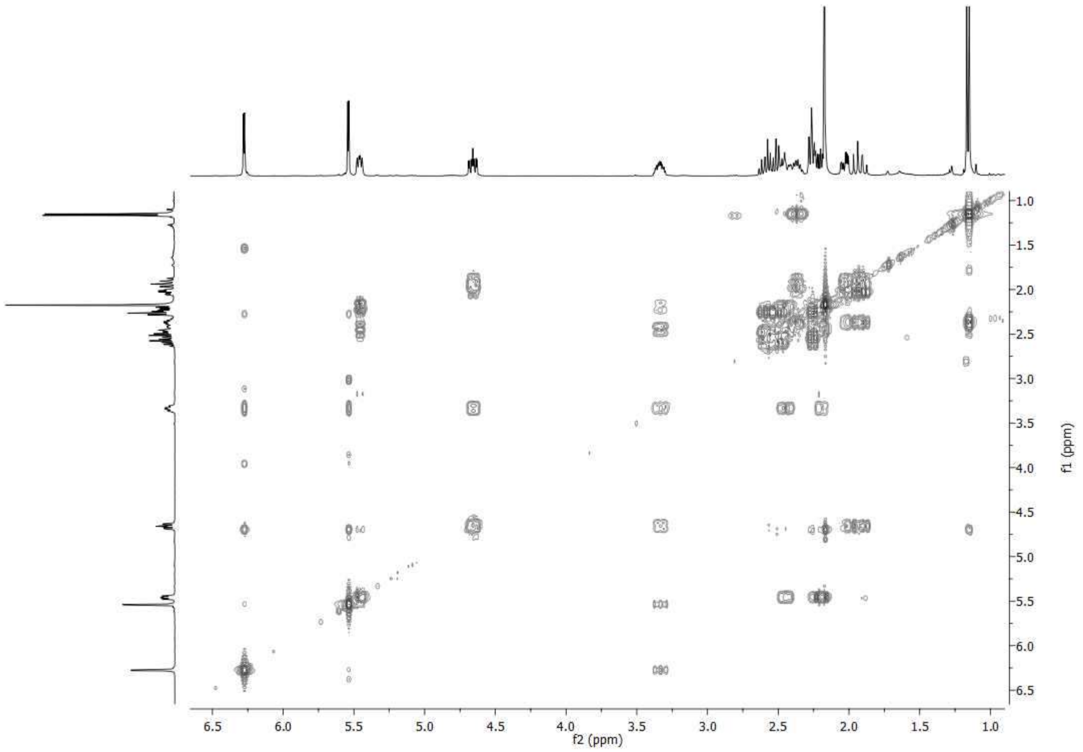

Spectra 5. COSY spectrum of inuloxin E (1) (CDCl3, 100 MHz).

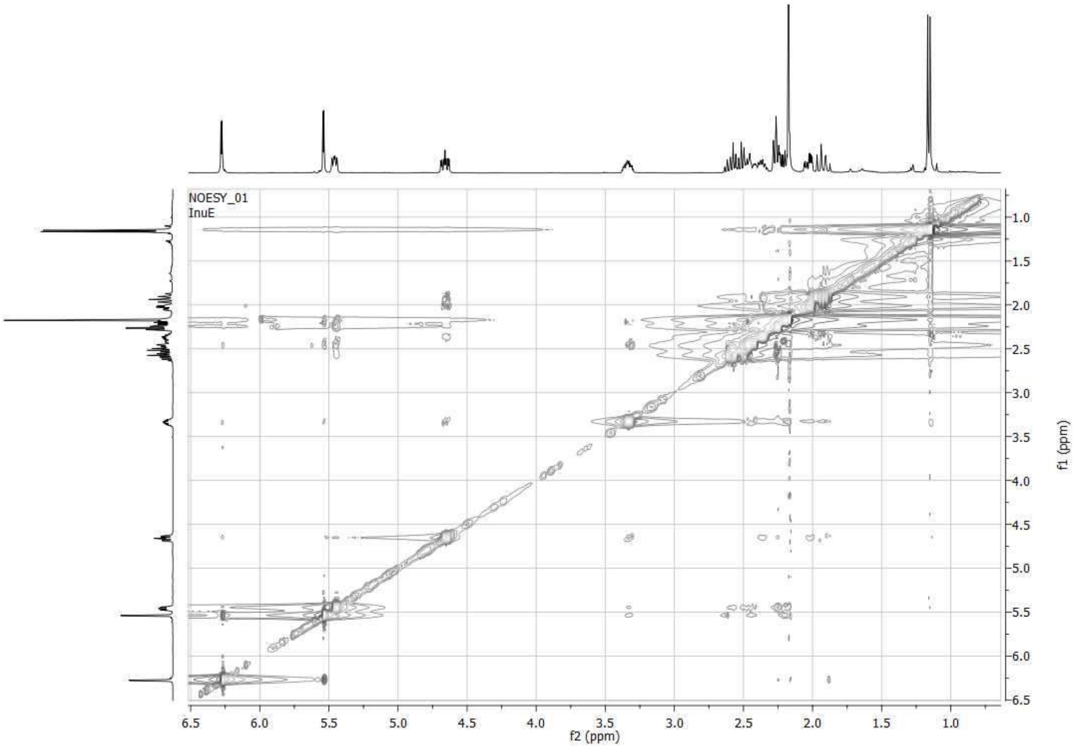

Spectra 6. NOESY spectrum of inuloxin E (**1**) ($CDCl_3$, 100 MHz).

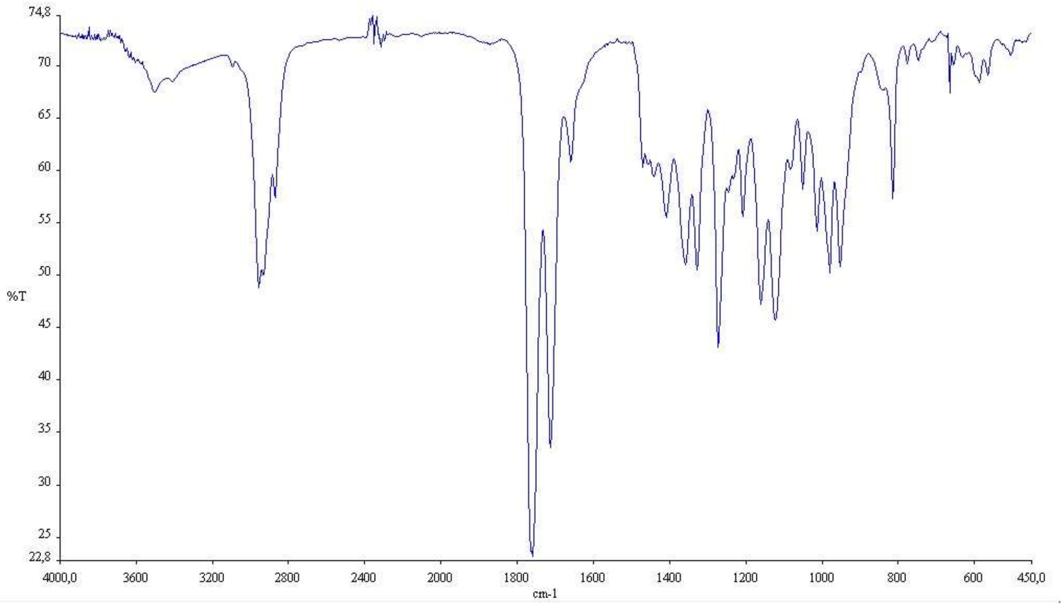

Spectra 7. IR spectrum of inuloxin E (**1**).
